# Extra-telomeric functions of telomerase in the pathogenesis of Epstein-Barr virus-driven B-cell malignancies and potential therapeutic implications

**DOI:** 10.1186/s13027-018-0186-5

**Published:** 2018-04-10

**Authors:** Silvia Giunco, Maria Raffaella Petrara, Manuela Zangrossi, Andrea Celeghin, Anita De Rossi

**Affiliations:** 1 Immunology and Molecular Oncology Unit, Istituto Oncologico Veneto (IOV)-IRCCS, Padova, Italy; 2 Department of Surgery, Oncology and Gastroenterology, Section of Oncology and Immunology, University of Padova, Padova, Italy

**Keywords:** Telomerase, TERT extra-telomeric functions, Epstein-Barr virus, Latent/lytic viral cycle, B-cell malignancies

## Abstract

The Epstein-Barr virus (EBV) is a ubiquitous human γ-herpesvirus causally linked to a broad spectrum of both lymphoid and epithelial malignancies. In order to maintain its persistence in host cells and promote tumorigenesis, EBV must restrict its lytic cycle, which would ultimately lead to cell death, selectively express latent viral proteins, and establish an unlimited proliferative potential. The latter step depends on the maintenance of telomere length provided by telomerase. The viral oncoprotein LMP-1 activates TERT, the catalytic component of telomerase. In addition to its canonical role in stabilizing telomeres, TERT may promote EBV-driven tumorigenesis through extra-telomeric functions. TERT contributes toward preserving EBV latency; in fact, through the NOTCH2/BATF pathway, TERT negatively affects the expression of BZLF1, the master regulator of the EBV lytic cycle. In contrast, TERT inhibition triggers a complete EBV lytic cycle, leading to the death of EBV-infected cells. Interestingly, short-term TERT inhibition causes cell cycle arrest and apoptosis, partly by inducing telomere-independent activation of the ATM/ATR/TP53 pathway. Importantly, TERT inhibition also sensitizes EBV-positive tumor cells to antiviral therapy and enhances the pro-apoptotic effects of chemotherapeutic agents. We provide here an overview on how the extra-telomeric functions of TERT contribute to EBV-driven tumorigenesis. We also discuss the potential therapeutic approach of TERT inhibition in EBV-driven malignancies.

## Background

The Epstein-Barr virus (EBV) is a ubiquitous human γ-herpesvirus infecting more than 90% of the world’s population. Primary infection with EBV is often asymptomatic, but it can also manifest as infectious mononucleosis. Although EBV may infect various cell types, such as epithelial cells and T or Natural Killer cells, it preferably infects B lymphocytes, in which it establishes a lifelong asymptomatic latent infection. In immunocompromised individuals, EBV may cause a wide range of cancers, of both hematopoietic and epithelial origin, including Burkitt’s lymphoma (BL), Hodgkin’s lymphoma (HL), post-transplant lymphoproliferative disorders (PTLD), AIDS-associated lymphomas, and nasopharyngeal and gastric carcinomas [1].

Like other γ-herpesviruses, EBV has both lytic and latent cycles. Primary EBV infection occurs in the oropharynx, leading to productive lytic infection of B lymphocytes. EBV antigens promote immune recognition, inducing an EBV-specific immune response which controls viral infection in the immunocompetent host, and the viral lytic cycle triggers the death of the infected cells [2]. In tumor cells, EBV expresses various sets of latency-associated proteins with transforming properties. The most restricted form of EBV latency (‘latency I’), found in BL cells, is characterized by the selective expression of EBV nuclear antigen (EBNA)-1. A second latency program (‘latency II’), in which EBV expresses EBNA-1 and the three latent membrane proteins (LMP-1, LMP-2A, LMP-2B), is found in tumor cells of HL and nasopharyngeal carcinomas. The full set of EBV-encoded latency proteins (‘latency III’), including the six EBNAs (EBNA-1, − 2, −3A, -3B, -3C, and -leader protein or LP) and the LMPs proteins, is usually present in PTLD and AIDS-associated lymphomas [1, 3]. In addition to its latent proteins, EBV encodes small non-polyadenylated, non-coding double-strand RNAs, called EBV-encoded RNAs (EBER), which are expressed in all forms of latency and may contribute to viral pathogenesis [4]. The oncogenic potential of EBV is highlighted by its ability to immortalize B cells in vitro, generating continuously proliferating lymphoblastoid cell lines (LCLs). LCLs may constitute an in vitro model of EBV-driven malignancies expressing the latency III program.

While latency programs predominate in EBV-driven tumors, lytic reactivation may occur in a small fraction of infected cells, favoring the spread of the virus [5, 6]. Lytic reactivation, induced by endogenous or exogenous stimuli, is orchestrated by up-regulation of two EBV immediate-early genes, *BZLF1* and *BRLF1* [7]. As lytic infection promotes the death of EBV-infected cells both in vitro and in vivo, the lytic induction strategy has been suggested as potential therapy to induce EBV-dependent tumor cell killing [8–10]. Triggering EBV lytic replication may be particularly effective and therapeutically important, as EBV lytic proteins can activate antiviral prodrugs, such as ganciclovir (GCV) or radiolabeled nucleoside analogs, which further promote the death of infected cells and also prevent the release of infectious viruses [11, 12]. Thus, the combination of antivirals with lytic cycle inducers is emerging as a promising strategy for treating EBV-driven tumors [13–15].

The establishment of EBV latency programs promotes cell proliferation, inhibits apoptosis, blocks viral lytic replication, and ensures accurate and equal partitioning of the episomal viral genome to daughter cells [16]. However, expression of latent EBV proteins is not sufficient to immortalize EBV-infected cells entirely. As in other oncogenic viruses, a critical step for EBV-driven transformation is to overcome cellular senescence and acquire unlimited proliferative potential. This step depends on activation of mechanisms for telomere maintenance [17, 18]. Although it has been suggested that in newly EBV-infected B lymphocytes telomere length can be maintained by alternative lengthening of telomeres (ALT) [19], only EBV-positive cells with sustained telomerase activity become truly immortalized, and it has been demonstrated that most EBV-driven tumors, as well as established LCLs, are telomerase-positive. By contrast, telomerase-negative EBV-infected cells, although exhibiting a prolonged lifespan, eventually undergo cellular senescence and terminate their lifespan through telomere shortening [17, 18]. In addition to its canonical role in stabilizing telomeres, current evidence shows that telomerase reverse transcriptase (TERT), the catalytic component of telomerase, can promote EBV-driven tumorigenesis through extra-telomeric functions [20–23]. Here we review the cross-talk between telomerase and EBV which is essential for the viral oncogenetic process and discuss potential therapeutic implications.

### Telomere maintenance in EBV-infected cells: The canonical role of telomerase

Telomeres are specialized DNA structures located at the ends of chromosomes which are essential for stabilizing chromosomes by protecting them from end-to-end fusion and DNA degradation [24]. In human cells, telomeres are composed of (TTAGGG)n tandem repeats associated with telomere-binding proteins, the shelterin complex, which form a special T-loop-like structure, thus avoiding the ends of chromosomes being recognized as double-strand DNA damage [25]. The progressive loss of telomeric repeats, which occurs at each round of DNA replication due to the inability of DNA polymerase to replicate the 3′ end of chromosomes completely [26], reduces the length of telomeres to a critical size. Such critically short telomeres are no longer protected by the shelterin complex and are recognized as DNA double-strand breaks which trigger the DNA damage response (DDR), resulting in cellular senescence and apoptosis [25]. To circumvent replicative senescence and acquire the ability to sustain unlimited replicative potential, tumor cells must stabilize their telomeres.

Although EBV-infected B cells exhibit higher proliferative activity than resting primary B lymphocytes, very few EBV-carrying B cells eventually progress to immortalization: most of them reach a proliferative crisis and end their lifespan after about 150 population-doubling levels, according to genetic factors, including telomere length. Soon after EBV infection, B lymphocytes may exhibit multiple signs of telomere dysfunction and ALT markers, including highly heterogeneous telomeres, appearance of extra-chromosomal telomeric DNA, accumulation of telomere-associated promyelocytic leukemia nuclear bodies, and telomeric-sister chromatid exchange [19]. This phenotype seems to be associated with EBV-mediated displacement of shelterin proteins and uncapping problems at telomeres, which may favor the activation of the ALT mechanism. ALT is an inherently imprecise recombination-based mechanism which may fuel the chromosomal and genomic instability that characterize newly established LCLs [19, 27]. However, only LCLs developing strong telomerase activity overcome cellular crises and become stably immortalized [17, 18]. Established LCLs with sustained telomerase activity show minimal or no signs of telomere dysfunction [19], thus revealing the prominent role of telomerase activation in ensuring telomere integrity during EBV immortalization.

Telomerase is a ribonucleoprotein complex containing an internal RNA template (telomerase RNA component, TERC) and a catalytic protein, TERT, with telomere-specific reverse transcriptase activity. TERT, which synthesizes de novo telomere sequences using TERC as a template, is the rate-limiting component of the telomerase complex, and its expression is correlated with telomerase activity. Although TERC has broad tissue distribution and is constitutively present in normal and tumor cells, the expression of TERT is usually repressed in normal somatic cells and is essential for unlimited cell growth, thus playing a critical role in tumor formation and progression [28].

Regulation of telomerase operates at several levels: transcription, mRNA splicing, subcellular location of each component, and assembly of TERC and TERT in an active ribonucleoprotein. Transcription of the TERT gene is probably the key determinant in regulating telomerase activity, since TERT transcription is specifically up-regulated in cancer cells but silent in most normal ones. The TERT promoter reveals complex regulation dynamics, whereby multiple transcriptional regulatory elements play functional roles in different contexts, either individually or interactively. TERT contains recognition sequences for many important transcription factors such as TP53, P21, SP1, ETS, E2F, AP-1, HIF1A and MYC [29]. Regulation of TERT transcription may also involve DNA methylation, as the TERT promoter contains a cluster of CpG sites [29]. Somatic mutations in the promoter of the TERT gene, which increase gene expression by creating de novo binding sites for the ETS/TCF transcription factors, have also recently been described [30]. At post-transcriptional level, more than 20 different TERT variants have been reported, some of which probably play critical roles in regulating telomerase activity [31]. Telomerase activity is also controlled by post-translational modifications of the TERT protein. Phosphorylation of the protein at critical sites along the PI3K/AKT kinase pathway seems to be crucial for telomerase activity and nuclear localization. Active recruitment of telomerase to telomeres is a necessary regulatory step and involves telomere-associated shelterin proteins [32].

Studies aimed at defining the mechanism underlying EBV-induced telomerase activation have demonstrated that LMP-1, the major EBV oncoprotein, up-regulates telomerase activity both in epithelial cells [33, 34] and in B lymphocytes [35, 36]. In particular, it has been demonstrated that LMP-1 activates TERT in nasopharyngeal carcinoma cells through the AKT pathway [34]: in established LCLs, LMP-1 activates TERT at transcriptional level *via* the NF-κB and MAPK/ERK1/2 pathways [36]. Of interest, while in epithelial cells TERT expression is also MYC-dependent and the mutagenesis of MYC-responsive E-box elements in the TERT promoter inhibits TERT transactivation by LMP-1 [33], in B cells TERT activation by LMP-1 is MYC-independent. In fact, mutagenesis in NF-κB binding sites, but not in MYC ones, inhibits LMP-1-transactivation of the TERT promoter [36]. This is of particular interest, since in most EBV-driven tumors, like the immunoblastic lymphomas occurring in AIDS patients and early PTLD lesions, *MYC* is in a germ-line configuration. In these malignancies, LMP-1 probably plays an essential role in TERT activation.

### Role of TERT in switch of latent/lytic cycle of EBV

The canonical explanation for the tumor-promoting role of telomerase is that it allows cells to escape the barrier to unlimited replicative potential caused by telomere attrition. Accumulating evidence suggests that, besides its canonical role in stabilizing telomeres, TERT also has other biological functions, including enhancement of cell proliferation, resistance to apoptosis, and regulation of DDR, [37–39]. TERT can also alleviate levels of cellular reactive oxygen species (ROS) by enhancing cellular antioxidant defense systems, thus allowing cancer cells to evade death stimuli [40] and can stimulate the epithelial-mesenchymal transition and induce stemness [41, 42].

The extra-telomeric roles of TERT have also been described in EBV-driven lymphomagenesis. TERT plays a critical role in establishing EBV latency and preventing the EBV lytic cycle, thereby contributing to transformed phenotypes. In particular, high levels of endogenous TERT or ectopic TERT expression in TERT-negative EBV-infected B cells prevents the induction of the viral lytic cycle. By contrast, TERT silencing by specific siRNA or short-hairpin (sh)RNA induces the expression of BZLF1, EA-D and gp350 EBV lytic genes, and triggers a complete lytic cycle. This occurs in both EBV-immortalized and fully transformed B cells, thus supporting the concept that TERT is a critical regulator of the balance between viral latent and lytic cycles [20, 21]. The treatment of primary EBV-positive BL with zidovudine (AZT), a thymidine analog, has also been demonstrated to induce the EBV lytic cycle and cell death through the NF-κB pathway [43, 44]. As AZT may inhibit telomerase activity [45], this finding further supports the close relationship between TERT activity and the EBV latent/lytic cycle.

Studies aimed at defining the mechanism(s) by means of which TERT prevents the viral lytic cycle have demonstrated the involvement of the NOTCH2/BATF pathway. BATF is a transcription factor expressed in hematopoietic tissues and in B cells infected with EBV [46–48]. In LCL, BATF is a critically important survival factor being involved in the suppression of pro-apoptotic BIM and in the induction of MYC [48]. Notably, BATF has been shown to inhibit the expression of BZLF1, thus reducing EBV lytic replication in latently infected B cells [47]. Of interest, BATF is a target gene of the NOTCH signaling pathway in B cells [47]. High expression of TERT in LCLs has been shown to activate NOTCH2 at transcriptional level through the NF-kB pathway; in turn, NOTCH2 activates BATF, which negatively affects the expression of BZLF1, thus repressing the EBV lytic program [22]. Accordingly, pharmacological inhibition of NOTCH2 signaling by γ-secretase inhibitors decreases canonical NOTCH target genes expression, including BATF, with a concomitant increase in early and late EBV lytic genes, and thus triggers a complete lytic cycle in both LCL and EBV-positive BL cells [22].

More recently, the impact of TERT on EBV infection and viral gene expression has also been studied in epithelial cells [49]. Gastric carcinoma AGS cells with high telomerase activity show increased expression of latent EBV genes, indicating that telomerase directly contributes toward favoring the latency program in epithelial EBV-infected cells [49]. Thus, the ability of TERT to favor the latency program in both B lymphocytes and epithelial EBV-infected cells further supports the crucial role of telomerase in EBV-driven malignancies.

### TERT inhibition as a therapeutic approach for EBV-driven malignancies

Telomerase inhibitors remain an attractive approach to target cancer cells, given the specificity of TERT expression in tumor cells. However, in theory, the time to antineoplastic effectiveness of telomerase inhibitors depends on the original length of the telomeres in cancer cells and, apparently, these agents are effective in halting tumor growth only after the cancer cells have shortened their telomeres. This aspect acquires particular importance in the context EBV-carrying malignancies as it has been demonstrated that EBV-positive BL cell lines show longer telomeres compared to EBV-negative BLs [50] and, during the early phases of EBV-induced growth of primary B cells, their telomeres length remain constant or even increase [19, 27, 51].

In this scenario, the evidence of extra-telomeric functions of TERT in cellular kinetics and resistance to apoptosis has recently opened the door to potential telomere length-independent therapeutic effects. In EBV-driven malignancies, besides sustaining the latency program required for the EBV-driven transformation, TERT may promote EBV tumor progression by enhancing the kinetics of cell proliferation. In fact, EBV-infected B cells with sustained telomerase activity grow faster than telomerase-negative cells [20]. Accordingly, TERT inhibition results in an anti-proliferative effect, inducing an accumulation of cells in the S phase, probably due to dephosphorylation of 4E-BP1, an AKT1-dependent substrate, which results in the decreased availability of proteins needed for cell cycle progression [21]. Thus, by slowing proliferation kinetics, TERT inhibition may represent an appealing suppressor strategy of EBV tumor growth.

As mentioned previously, the first mechanism by means of which short-term inhibition of TERT may induce cell death of EBV-transformed cells is induction of the EBV lytic cycle. Inhibition of TERT leads to down-regulation of BATF and up-regulation of BZLF1, the main regulator of the viral lytic cycle. Notably, cell death induced by TERT inhibition in EBV-positive cells does not depend only on the induction of the EBV lytic cycle: inhibition of TERT with short hairpin (sh)RNA in both EBV-positive and EBV-negative BL cell lines induces apoptosis via a AKT1/FOXO3/NOXA pathway [21]. In particular, TERT silencing induces inhibition of AKT1 kinase, which is associated with dephosphorylation/activation of the transcription factor FOXO3, an effector of AKT1 kinase functioning in several cell activities, including survival. In turn, FOXO3 induces up-regulation of NOXA, a pro-apoptotic protein, the expression of which is known to be blocked by latent EBV infection [52]. Thus, TERT inhibition can overcome the block of NOXA up-regulation induced by EBV, favoring cell apoptosis [21]. Notably, although pharmacological inhibition of AKT1 does not reveal any evidence of EBV lytic replication in EBV-positive B cells, thus indicating that the EBV lytic cycle induced by TERT inhibition occurs via an AKT1-independent pathway [21], the pharmacological inhibition of NOTCH2 triggers the EBV lytic cycle, thus confirming the critical involvement of the NOTCH2, BATF and BZLF1 pathways in the latent/lytic EBV cycle [22].

In EBV-positive tumor cells, the lytic cell cycle induced by TERT inhibition may be exploited to sensitize cells to antiviral drugs such as GCV. GCV is an antiviral prodrug activated by EBV lytic protein kinase [10, 11]. Phosphorylated/activate GCV competitively inhibits both viral and cellular DNA synthesis, thus resulting in both cell death of infected cells and reduction of viral replication [8]. Consequently, GCV markedly enhances the anti-proliferative and pro-apoptotic effects of TERT inhibition in both EBV-positive LCLs and BL [21]. Thus, the combination of antiviral drugs with strategies capable of inhibiting TERT expression/activity may result in therapeutically substantial effects in patients with EBV-related malignancies. Consistently, as EBV lytic reactivation after TERT inhibition is mediated by the NOTCH2/BATF pathway, GCV also enhances the apoptotic effect of γ-secretase inhibitors which, by blocking NOTCH2/BATF signaling, trigger viral lytic reactivation [22].

Most recent data show that LCLs and both EBV-positive and EBV-negative BL cells short-term treated with BIBR1532 (BIBR), a chemical compound which selectively inhibits the catalytic activity of TERT [53], undergo cell cycle arrest in S phase and apoptosis [23]. These effects are telomerase-specific and have not been observed in telomerase-negative cell lines. The cell cycle arrest and apoptosis subsequent to TERT inhibition are associated with and probably dependent on activation of the DDR pathway. TERT inhibition does induce DDR, highlighted by increased levels of γH2AX, activation of ATM and ATR and their downstream substrates CHK1, CHK2 and pro-apoptotic TP53. Notably, the DDR pathway activated after short-term exposure to BIBR is not related to telomere dysfunction, as BIBR treatment does not affect the mean and range of telomere lengths and γH2AX damage foci are randomly diffuse, rather than being specifically located on telomeres [23]. It has been demonstrated that the productive cycle of EBV elicits ATM-dependent DDR, and provides an S-phase-like cellular environment suitable for viral lytic replication [54]. Thus, it is reasonable that, in EBV-positive background, cell cycle arrest in S phase and DDR activation consequent upon TERT inhibition is partly orchestrated by the induction of the lytic cycle.

In addition, treatment of LCL with BIBR in combination with Fludarabine or Cyclophosphamide, two agents frequently employed to treat B-cell malignancies, induces a significant increase in specific cell death compared with results seen after treatment with chemotherapeutic agents alone. These results may lead to the substantial clinical application of TERT inhibitors in combination with standard chemotherapeutic protocols to treat EBV-positive B-cell malignancies [23].

## Conclusions

**Fig. 1:**
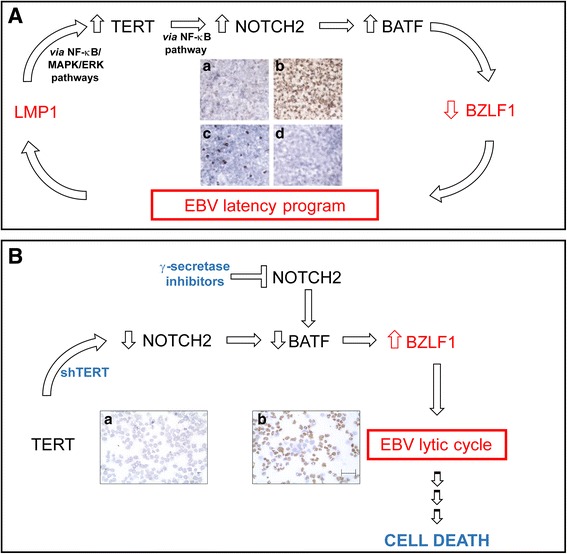
TERT levels affect EBV latent/lytic status. **A**, cross-talk between EBV and TERT to sustain viral latency program: in EBV-infected primary **B** lymphocytes, activation of TERT occurs concomitantly with induction of latent EBV proteins and down-regulation of EBV lytic gene expression. EBV-encoded LMP-1 activates TERT at transcriptional level *via* NF-κB and MAPK/ERK1/2 pathways. In turn, TERT expression activates NOTCH2 at transcriptional level *via* NF-κB pathway. NOTCH2 activates BATF, which negatively affects the expression of BZLF1, a master regulator of viral lytic cycle, thus favouring induction and maintenance of EBV latency program, essential for EBV-driven transformation. Immunohistochemical image: TERT (*a*, *b*) and BZLF1 (*c*, *d*) protein expression in early- (*a*, *c*) and late- (*b*, *d*) infected B lymphocytes (X40). B, TERT or NOTCH inhibition triggers EBV lytic cycle: TERT silencing by shRNA (shTERT) or inhibition of NOTCH signaling by γ-secretase inhibitors lead to NOTCH2-dependent down-regulation of BATF and up-regulation of BZLF1, inducing a complete EBV lytic cycle. Immunohistochemical image: EBV lytic gp350 protein expression in EBV-positive BL cells untreated (*a*) and treated (*b*) for 72 h with shTERT (X20). Scale bar, 100 μm. See the text for details

Since a latent program is required to promote EBV tumorigenesis, whereas the lytic cycle induces cell death, the finding that TERT, besides maintaining telomere integrity, also plays a critical role in the establishment of EBV latency and in preventing the EBV lytic cycle (Fig. 1A), supports the view that TERT inhibition is an appealing therapeutic strategy against EBV-driven malignancies. By triggering the viral lytic cycle, TERT inhibition induces cell death (Fig. 1B) and sensitizes EBV-infected cells to antiviral drugs. Notably, besides triggering the viral lytic cycle *via* the NOTCH2/BATF/BZLF1 pathway (Fig. 1B), short-term inhibition of TERT activates pro-apoptotic programs *via* the AKT1/FOXO3/NOXA and ATM/ATR/TP53 pathways. Notably, cell cycle arrest and the pro-apoptotic effects of short-term TERT inhibition are independent of telomere length. Thus, in both EBV-driven and virus-unrelated B-cell malignancies inhibition of TERT seems to be an effective approach in inducing cell death, regardless of telomere length. In vitro experiments also demonstrate that the therapeutic approach based on inhibition of TERT enhances the pro-apoptotic and anti-proliferative effects of chemotherapeutic agents in EBV-transformed cells. Further studies of primary tumor cells from patients with EBV-driven malignancies and suitable animal models will pave the way for a solidly based pre-clinical rationale for including TERT inhibitors in chemotherapy protocols for treating these malignancies.

## Abbreviations

ALT: Alternative length of telomeres
AZT: Zidovudine
BIBR: BIBR1532
BL: Burkitt’s lymphoma
DDR: DNA damage response
EBER: EBV-encoded RNAs
EBNA: EBV nuclear antigen
EBV: Epstein-Barr virus
GCV: ganciclovir
HL: Hodgkin’s lymphoma
LCL: Lymphoblastoid cell line
LMP: Latent membrane protein
PTLD: Post-transplant lymphoproliferative disorders
ROS: Reactive oxygen species
TERC: Telomerase RNA component
TERT: Telomerase reverse transcriptase
